# Comparative analysis to investigate a possible mechanism for cell enlargement in succulent leaves of *Crassothonna capensis* (*Asteraceae*)

**DOI:** 10.1007/s10265-025-01674-0

**Published:** 2025-11-11

**Authors:** Hokuto Nakayama, Kento Sawazaki, Yuki Doll, Hiroyuki Koga, Huibo Yu, Yasutake Moriyama, Mikita Tamura, Hirokazu Tsukaya

**Affiliations:** Department of Biological Sciences, The University of Tokyo, Science Build. #2, 7-3-1, Hongo, Bunkyo-Ku, Tokyo, 113-0033 Japan

**Keywords:** Asteraceae, *Crassothonna*, Endoreduplication, Leaf development, Succulent leaves, Succulent plants

## Abstract

Succulent plants, characterized by the presence of water-storage tissues, often exhibit distinctive leaf morphology. However, their developmental mechanisms remain largely unknown, partly due to the lack of an appropriate model plant. In this study, we evaluated the potential of Asteraceae species as a model system for investigating the mechanisms underlying leaf succulence. First, we analyzed the leaf anatomical and cellular characteristics of succulent plants in the genera *Caputia*, *Crassothonna*, *Curio*, *Othonna*, and *Senecio*. To explore a potential mechanism involved in succulent leaf development, we focused on endoreduplication—genome replication without mitosis—and measured the ploidy levels of leaf cells in each species using flow cytometry (FCM) to assess the relationship between leaf succulence and endoreduplication. The FCM data indicated that succulent leaves of *Caputia*, *Curio*, *Senecio*, and *Othonna* were not associated with endoreduplication. In contrast, endoreduplication was detected in enlarged leaf cells of *Crassothonna capensis*, while no endoreduplication was observed in the peduncles, which did not appear succulent, or in ligules, which are the lateral organs homologous to leaves. These results suggest that unknown mechanisms other than endoreduplication contribute to leaf succulence in certain genera, and that endoreduplication is regulated in an organ-specific manner in *Cr. capensis*. Additionally, even if endoreduplication is involved in leaf succulence, it may serve as a supplementary mechanism for cell enlargement. Collectively, these findings highlight *Crassothonna* and its related genera in Asteraceae as a promising group for studying the mechanisms of leaf succulence.

**Supplementary Information:**

The online version contains supplementary material available at 10.1007/s10265-025-01674-0.

## Introduction

Succulent plants are characterized by the presence of water-storage tissues that allow them to be temporarily independent of the external water supply (Males 2017). Succulence is an adaptation to low water availability, and it includes many primary and secondary functions (e.g., short-/long-term water storage, salt accumulation, and thermal insulation) that reduce water stress (Males 2017). Besides leaves and stems, succulence can occur in any vegetative organ, such as the bulbs or tubers of geophytes, orchid pseudobulbs, and the parenchymatous rays of pachycauls (Eggli and Nyffeler 2009; Males 2017). Succulents are found worldwide and approximately 3–5% of all angiosperms are commonly described as succulents (Griffiths and Males 2017). Moreover, succulence also occurs in some epiphytic ferns and gymnosperms (Ogburn and Edwards 2010), indicating that the succulent syndrome has evolved independently across land plants. These succulent plants have long drawn the attention of plant biologists because of the uniqueness of this phenomenon, and numerous studies have been conducted on the morphological, physiological, and ecological aspects of succulence (Hearn et al. 2013; Kaul 1977; Leverett et al. 2023; Ogburn and Edwards 2010; Ripley et al. 2013). A previous study has classified this wide range of succulence into two types based on anatomical and cellular characteristics: ‘all-cell succulence’ and ‘storage succulence’. In all-cell succulents, all cells perform photosynthesis and store water, whereas in storage succulents there is a functional demarcation, with chlorenchyma cells performing photosynthesis and hydrenchyma cells storing water (Griffiths and Males 2017; Ihlenfeldt 1985). A study using succulent leaves of Aizoaceae has revealed that the degree of leaf succulence was positively correlated with an enhanced ability to buffer photosynthetic capacity and maintain light reaction stability, even under water stress (Ripley et al. 2013). A more recent study has indicated differences in cell wall biochemistry between succulent and non-succulent leaves, pointing to the existence of a ‘glycomic diversity’ as shown by glycomic features seen in certain succulent lineages (Fradera-Soler et al. 2022). Additionally, some succulent plants exhibit endoreduplication which is a genome replication in the absence of mitosis (De Rocher et al. 1990; Powell et al. 2020). Notably, this endoreduplication is involved in cell enlargement in certain tissues of some plant species, for instance, in epidermal pavement cells in *Arabidopsis thaliana* (Arabidopsis) (Galbraith et al. 1991; Gendreau et al. 1997; Hülskamp et al. 1999; Katagiri et al. 2016; Kawade and Tsukaya 2017; Melaragno et al. 1993). Therefore, succulence represents a compelling subject of research across various scientific disciplines. Despite numerous studies (Heyduk 2021; Nakayama 2024), the developmental mechanisms underlying succulent tissue formation remain poorly understood, primarily due to the absence of an appropriate model plant. Establishing such a model system would be instrumental in elucidating these mechanisms.

In the present study, we used succulent plants of the genera *Caputia*, *Crassothonna, Curio*, *Othonna*, and *Senecio* in Senecioneae within Asteraceae to survey the possibility as a model system. *Curio* and *Crassothonna* were recently separated from *Senecio* and *Othonna*, respectively (Fig. S1) (Cicuzza et al. 2018; Nordenstam 2012). Although plants in the genus *Senecio* show a wide range of leaf morphological and anatomical variations (e.g., flat vs. terete, presence or absence of succulence), plants in the genus *Curio* are characterized by terete leaves with clearly defined tissue succulence (Cicuzza et al. 2018; Pelser et al. 2007).

**Fig. 1:**
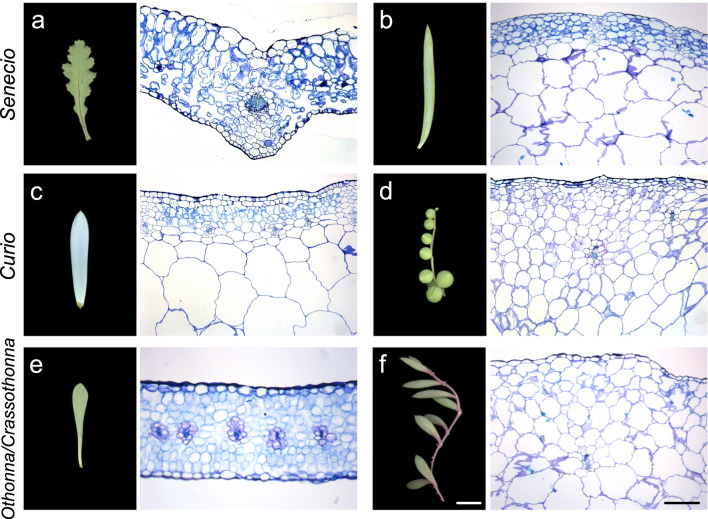
Comparison of gross leaf morphology and inner tissue anatomy. **a** *Senecio vulgaris*. **b** *Senecio antandroi.* **c** *Curio repens*. **d** *Curio rowleyanus*. **e** *Othonna euphorbioides*. **f** *Crassothonna capensis*. Left: gross morphology; right: inner morphology. All leaf pictures have the same magnification, and all sections have the same magnification. The top of the section image is the adaxial side. White bar = 1 cm; Black bar = 200 µm

Similarly, plants in *Othonna* exhibit variations in leaf type, including species with fleshy leaves (Magoswana et al. 2019), while the sister genus *Crassothonna* consists of 13 species with distinct terete succulent leaves (Nordenstam 2012) and both genera are members of the subtribe Othonninae, within Senecioneae. Plants in the genus *Caputia* are succulent perennial herbs and shrubs with more or less fleshy leaves. Previous studies suggest that the genus *Caputia* seems to diverge earlier than *Curio*, *Crassothonna*, *Othonna*, and *Senecio* (Kandziora et al. 2016) (Fig. S1). These facts make these genera suitable for comparative studies on the mechanisms underlying leaf succulence (Fig. 1). First, using these plants, we performed several anatomical analyses and then monitored ploidy levels of leaf cells by flow cytometry (FCM) to address the relationship between leaf succulence and endoreduplication.

## Materials and methods

### Preparation and observation of plant samples

The succulent plants used in this study were cultivated in a greenhouse on the Hongo campus of the University of Tokyo, Japan, before sample collection. All plants used in this study were purchased from nurseries as listed in Table S1. Plants were planted in pots containing soil and watered daily and every 2–3 d during summer and winter, respectively. Fully-expanded leaves were imaged using an Epson GT-X830 Photo scanner (Epson, Suwa, Japan). Mature leaf, ligule, and peduncle were collected and fixed in formalin acetic acid-alcohol (FAA) fixative (2.5% formalin, 2.5% acetic acid, and 45% ethanol [v/v]). Because *Crassothonna capensis* was the most readily available and easy to cultivate among *Crassothonna* plants used in this study, it was used in subsequent analyses using ligules and peduncles. These were used as controls since they were not succulent organs, in comparison to the mesophyll cells. The fixed samples were dehydrated by treating them with a graded series of ethanol (from 50 to 100% [v/v]) and subsequently embedded in Technovit 7100 resin (Kulzer and Co., Wehrheim, Germany). The embedded samples were sectioned into 8- to 10-µm-thick sections using an HM360 rotary microtome (Thermo Fisher Scientific, Waltham, MA, USA), stained with 0.02% (w/v) toluidine blue in phosphate-buffered saline, and then rinsed with water. Sections were mounted onto glass slides with Entellan New Mounting Medium (Merck, Darmstadt, Germany). Hand-cut sections of fresh leaves were placed in water. All sections were visualized using a light microscope (DM4500; Leica Microsystems GmbH, Wetzlar, Germany). Fully expanded mature organs were used for all the analyses. Plants used for the observation were listed in Table S1.

### Cell size comparison

Images of the sections were used to measure epidermal and inner cell sizes in leaves, ligules, and peduncles. Cells in the outermost layer of each organ and fully expanded inner cells were selected as the epidermal and inner cells, respectively. Cell areas were calculated using Fiji (Schindelin et al. 2012) (ImageJ2; ver. 2.9.0). To calculate cell area, six epidermal and inner cells in an image of the section were used. The experiments were performed three times with independent individuals (*n* = 18 from 3 individuals). Plants used for the comparison are listed in Table S1.

### Flow cytometry (FCM)

FCM was performed as previously described (Kozuka et al. 2005) with certain modifications. Mature leaves, ligules, and peduncles were chopped into pieces using a razor in nucleus extraction buffer [10 mM Tris–HCl pH 8.0 (Nacalai Tesque Inc., Kyoto, Japan), 2 mM MgCl_2_ (Nacalai Tesque Inc., Kyoto, Japan), 50 mg/L, 30 g/L Polyvinylpyrrolidone K30 (Nacalai Tesque Inc., Kyoto, Japan), 0.1% (v/v) Triton-X100 (Nacalai Tesque Inc., Kyoto, Japan)] and incubated for ≥ 15 min on ice to extract nuclei. The buffer was filtered using a 30 µm filter and was subsequently mixed with propidium iodide (Sigma-Aldrich, St. Louis, MO, USA) (ca. 20 mg/L in final concentration) to stain nuclei. The stained nuclei solution was analyzed using Accuri C6 (BD Bioscience, San Jose, CA, USA). The experiments were performed three times with independent individuals (*n* = 3). The most representative data are shown in the figure. Plants used for FCM are listed in Table S1.

## Results

### Types of leaf succulence in genera *Crassothonna, Curio*, *Othonna*, and *Senecio*

To investigate the types of succulents observed in Asteraceae, we focused on the distribution of chlorenchyma and hydrenchyma in leaves, as the two types of succulents are defined by their distribution (Griffiths and Males 2017). No distinct enlarged cells were observed in *Senecio vulgaris* L., a species with flattened and non-succulent leaves (Figs. 1a and S2). However, clear differentiation of the chlorenchyma and hydrenchyma was observed in the succulent leaves of *Senecio antandroi* Scott Elliot (Figs. 1b and S2), indicating that this species is a storage succulent. Similarly, the succulent leaves of *Curio repens* (L.) P.V.Heath and *Curio rowleyanus* (H.Jacobsen) P.V.Heath also showed distinct chlorenchyma and hydrenchyma (Figs. 1c, d and S2), indicating that these are storage succulent species. Next, we examined the leaf anatomy of *Othonna euphorbioides* Hutch. and *Crassothonna capensis* (L.H.Bailey) B.Nord. *O. euphorbioides* had flattened and fleshy leaves, and no enlarged cells were observed (Figs. 1e and S2). Succulent leaves of *Cr. capensis* showed enlarged cells; however, in hand sections, a clear achlorophyllous center of the leaf was observed (Figs. 1f and S2). Hence, this is a storage succulent species. In *Caputia scapose*, distinct enlarged cells and differentiation of the chlorenchyma and hydrenchyma were observed in the succulent leaves (Fig. S3).

### The leaves of *Crassothonna, Curio*, *Othonna*, and *Senecio* species show a wide range of cell enlargement

**Fig. 2:**
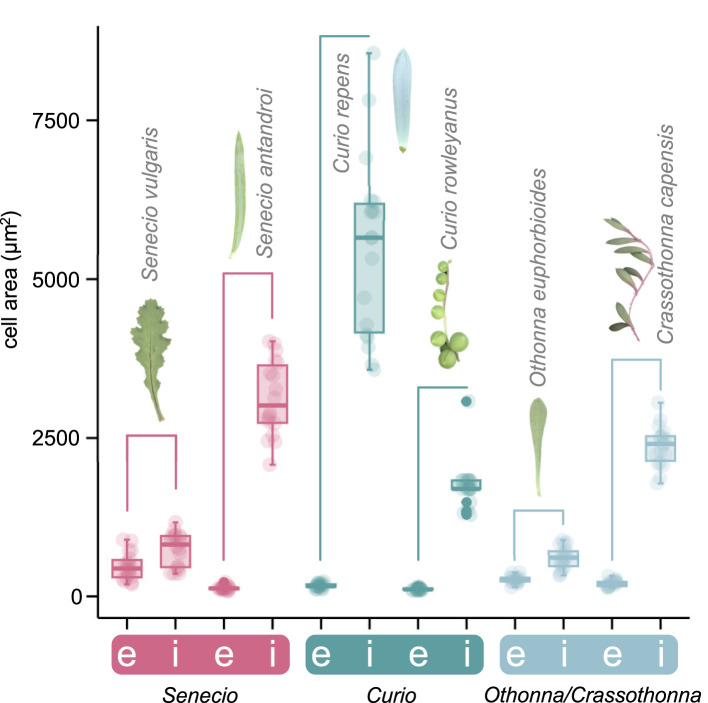
Comparison of leaf cell size in Senecioneae and Othonninae species. Area of epidermal and inner cells in mature leaves (*n* = 18 from 3 individuals). e: epidermal cells; i: inner cells. Horizontal bars indicate comparisons between two datasets obtained from the same plant species

We measured cell size in mature leaves to compare the anatomy of succulent and non-succulent species. In all species examined, epidermal cells were consistently smaller than internal cells, and their size remained relatively constant (Fig. 2). In contrast, in *S*. *antandroi*, a succulent species, the internal leaf cells were larger than those in *S. vulgaris* (Fig. 2). Species in the genus *Curio*, which is closely related to *Senecio* and classified as succulent, also exhibited enlarged internal leaf cells (Fig. 2). Although the internal leaf cells in the flattened leaves of *O. euphorbioides* were only slightly larger than the epidermal cells, those in *Cr*. *capensis*, a succulent species from a closely related genus, were much larger (Fig. 2). These findings suggest that species in the genera *Crassothonna*, *Curio*, *Othonna*, and *Senecio* provide a valuable comparative system due to the broad variation in leaf cell enlargement observed among them.

### Succulent leaves of *Crassothonna* species show endoreduplication

**Fig. 3:**
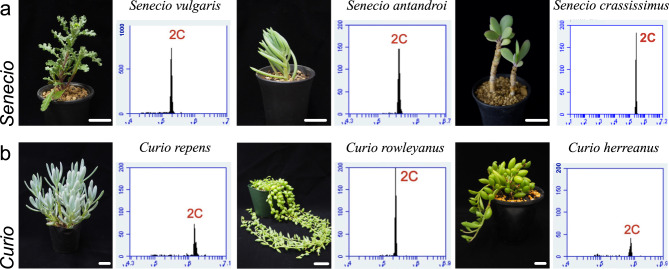
Comparison of nuclear ploidy distribution in leaves of *Senecio* and *Curio*. Nuclear ploidy distribution in *Senecio* (**a**: top) and *Curio* (**b**: bottom). Left, side view of the shoot; right, nuclear ploidy distribution in leaves. The x-axis indicates the signal intensity of propidium iodide, which reflects the nuclear DNA content and the y-axis indicates the cell counts. Bars = 2 cm

**Fig. 4:**
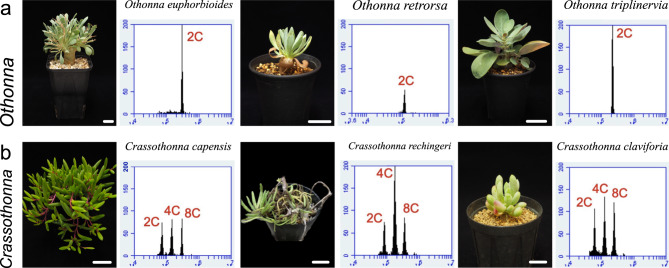
Comparison of nuclear ploidy distribution in leaves of *Othonna* and *Crassothonna*. Nuclear ploidy distribution in *Othonna* (**a**: top) and *Crassothonna* (**b**: bottom). Left, side view of the shoot; right, nuclear ploidy distribution in leaves. The x-axis indicates the signal intensity of propidium iodide, which reflects the nuclear DNA content and the y-axis indicates the cell counts. Bars = 2 cm

Then, FCM was performed to investigate the association between endoreduplication and cell enlargement in succulent leaves. First, we determined the ploidy levels (chromosomal copy number: C) of leaves of non-succulent *S. vulgaris,* and a single peak was observed, which indicated a ploidy level of 2C. Similarly, the ploidy levels of leaf cells in *S. antandroi* and *Senecio crassissimus* in the genus *Senecio*, were also 2C (Fig. 3a). Additionally, we tested succulent leaf cells of the genus *Curio*, which are closely related to *Senecio*. The ploidy levels of all tested *Curio* species (*C*. *repens*, *C*. *rowleyanus*, and *C*. *herreanus*) were 2C (Fig. 3b). Therefore, endoreduplication was not observed in the genera *Senecio* and *Curio*, regardless of whether the leaves were succulent. Next, to test whether this trend was the same in other succulent Asteraceae species, we investigated the leaves of members of the genera *Othonna* and *Crassothonna*. The ploidy levels of flattened leaves in *O*. *euphorbioides*, *O*. *retrorsa*, and *O*. *triplinervia* were 2C, similar to those of *S. vulgaris* with flattened leaves (Fig. 4a). In contrast, the ploidy levels of succulent leaves in *Cr*. *capensis*, *Cr*. *rechingeri*, and *Cr. clavifolia* showed three distinct peaks, which were estimated at 2C, 4C, and 8C, respectively (Fig. 4b). Additionally, we examined the ploidy levels of leaf cells in *Caputia scaposa*, a succulent species in the genus *Caputia* which diverged earlier than other genera. The ploidy level was 2C (Fig. S3). Taken together, endoreduplication was not observed in *Senecio*, *Curio*, *Othonna,* or *Caputia,* regardless of whether the leaves were succulent. On the other hand, high ploidy levels were observed only in succulent leaves in the genus *Crassothonna*.

### Endoreduplication is associated with enlarged leaf cells in *Crassothonna capensis*

**Fig. 5:**
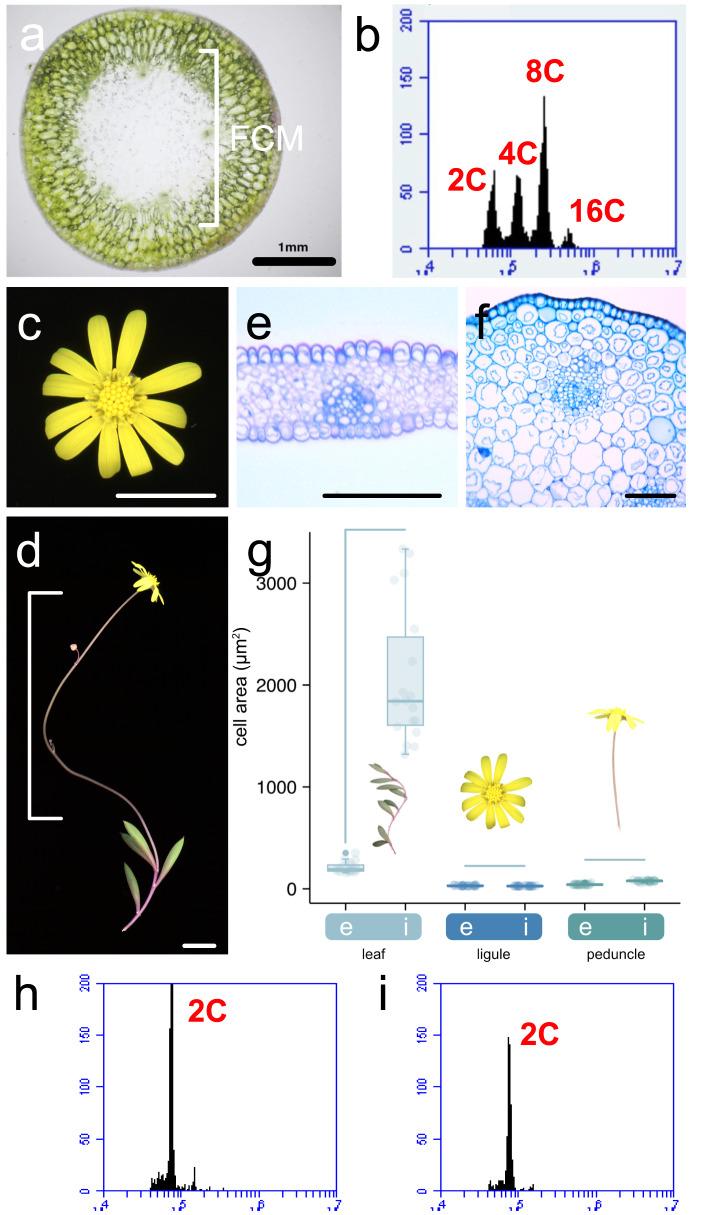
Nuclear ploidy distributions with leaf inner tissue, ligule, and inflorescence stem in *Crassothonna capensis*. **a** The inner morphology of a leaf. The white bracket shows the inner cells used for flow cytometry. **b** Nuclear ploidy distribution only with leaf inner cells. **c** Gross morphology of a capitulum. **d** Gross morphology of a peduncle. Inner tissue morphology of a ligule of a ligulate floret **e** and a peduncle **f**. **g** Area of epidermal and inner cells in each organ (*n* = 18 from 3 individuals). e: epidermal cells; i: inner cells. Horizontal bars indicate comparisons between two datasets obtained from the same plant species. Nuclear ploidy distribution with ligules **h** and a peduncle **i**. The x-axis indicates the signal intensity of propidium iodide, which reflects the nuclear DNA content and the y-axis indicates the cell counts. Bars = 1 mm in **a**, 1 cm in **c** and **d**, and 100 µm in **e** and **f**

To examine whether endoreduplication is specific to certain tissues or organs, we performed FCM using only the inner hydrenchyma cells from the leaves of *Cr. capensis*, in order to determine whether the enlarged leaf cells had undergone endoreduplication (Fig. 5a). The results showed multiple distinct peaks at 2C, 4C, 8C, and 16C (Fig. 5b). Next, FCM was performed using ligules, which are the same lateral organs as leaves. We also used peduncles that did not appear succulent (Fig. 5c, d). No distinct enlarged cells were observed in the cross-sections of a ligule or peduncle in *Cr. capensis* (Fig. 5e, f), and in both types of organs, the internal cell size was not dramatically larger compared to the epidermal cells (Fig. 5g). FCM revealed that the ploidy levels of the ligules and peduncles were 2C (Fig. 5h, i).

## Discussion

Not all enlarged cells are necessarily linked to increased DNA content/ploidy (Tsukaya 2019). For instance, in Arabidopsis, compensated cell enlargement, which is triggered by a decrease in cell proliferation in the leaves, is mostly independent of endoreduplication (Ferjani et al. 2007). In the present study, no association between leaf cell enlargement and endoreduplication has been observed in *Caputia*, *Curio*, and *Senecio*, all of which include succulent species. This suggests that in certain genera of Asteraceae, an as-yet unidentified mechanism, distinct from endoreduplication, may be responsible for inducing cell enlargement, thereby contributing to leaf succulence. Additionally, the absence of endoreduplication in the succulent leaves of *Caputia* suggests that a mechanism of cell enlargement independent of endoreduplication may represent an ancestral trait among the plant species examined in this study. Previous studies have shown that the Asteraceae, including the Senecioneae, underwent a rapid diversification process (Mandel et al. 2019; Panero and Crozier 2016). Although the specific details remain unknown, if succulence was achieved within the framework of existing leaf developmental mechanisms, with no major modifications, it may have contributed to adaptation to arid environments and may have been one of the factors that enabled these plants to evolve rapidly. If succulence arises from simple modifications of pre-existing developmental programs, this could represent one possible reason why the succulent syndrome has evolved multiple times across land plants. On the other hand, cases in which cell enlargement is associated with endoreduplication have also been reported. For instance, the size of leaf epidermal cells correlates well with endoreduplication levels in Arabidopsis (Melaragno et al. 1993), and a previous study suggested that the increase in cell size observed in polyploid plants is attributed to a mechanism similar to cell enlargement mediated by endoreduplication (Breuer et al. 2007). In *Cr*. *capensis*, no endoreduplication was detected in the ligules of ligulate florets, which are the lateral organs homologous to leaves, whereas distinct endoreduplication was detected in hydrenchyma cells. These findings indicate that endoreduplication may contribute to succulent leaf development in the genus *Crassothonna*, and, if so, that it is regulated at the organ level. Similar organ-level regulation of endoreduplication has been reported in plants of the Aizoaceae. However, in Aizoaceae, endoreduplication also occurs in floral organs, which differs from the case in *Crassothonna*. (Braun and Winkelmann 2016). This difference is considered to arise from independent acquisitions of succulence accompanied by endoreduplication across different lineages. Even if endoreduplication is involved in leaf succulence, its contribution may be limited. This is because, as mentioned earlier, in genera such as *Caputia* and *Curio*, leaf succulence occurs without endoreduplication, suggesting that its role may be supplementary-acting in coordination with other mechanisms. If it is not related to cell size control, endoreduplication may instead be associated with other physiological or ecological functions of *Crassothonna* leaves. This is because no distinctive morphological features that would require endoreduplication are observed in the leaves of *Crassothonna* when compared with those of other species. In that case, as suggested by a previous study, endoreduplication may be related to drought or salt tolerance (Ceccarelli et al. 2006; Tian et al. 2019).

Although the detailed mechanisms of leaf succulence remain unclear, it is highly likely that at least *Senecio* and *Curio* share the same mechanism. These two were originally classified within the same genus and are currently considered sister genera based on recent phylogenetic studies (Cicuzza et al. 2018). In contrast, *Othonna* and *Crassothonna,* which were split from the genus *Othonna,* do not appear to be sister genera to *Senecio* and *Curio* (Fig. S1) (Kandziora et al. 2016; Mandel et al. 2017; Pelser et al. 2010), suggesting that *Senecio*/*Curio* are phylogenetically distant from *Othonna*/*Crassothonna*. Therefore, if endoreduplication is involved in leaf succulence, the presence of an additional mechanism of cell enlargement only in *Crassothonna* may be explained by the fact that leaf succulence is a trait that has evolved independently throughout plant evolution. This indicates that the cell enlargement observed in the genus *Crassothonna* may be fundamentally different from that seen in other genera such as *Caputia*, *Curio*, *Othonna*, and *Senecio*. Of course, these evolutionary interpretations may require revision if more robust phylogenetic relationships among the relevant genera become elucidated. Altogether, the mechanisms of leaf succulence appear to differ among these genera, and there may be unknown mechanisms beyond endoreduplication.

## Conclusions

Although succulent plants have long fascinated researchers, the mechanisms underlying their cell enlargement remain unclear. In this study, we found that cell enlargement mechanisms are not associated with endoreduplication in genera such as *Caputia*, *Curio*, *Othonna*, and *Senecio*. In contrast, endoreduplication was observed in the genus *Crassothonna*, suggesting that, at least in the succulent leaves of Senecioneae examined in this study, endoreduplication is not the primary driver of cell enlargement and that, in the genus *Crassothonna*, endoreduplication may serve as a potential supplementary mechanism for cell enlargement in the succulent leaves. Therefore, if it is involved at all, its contribution appears to be restricted to a limited number of lineages. A more detailed analysis of *Cr. capensis* and its closely related species will provide a foundation for determining whether the observed association in *Crassothonna* is causal or correlative. Furthermore, a wide range of succulent leaf morphologies was observed, sometimes even within the same genus, tribe, or family. This diversity makes these genera particularly suitable for comparative studies aimed at elucidating the mechanisms underlying leaf succulence. Moreover, previous studies have shown that in *S*. *crassissimus*, the cell wall composition of succulent leaves differs from that of non-succulent plants (Fradera-Soler et al. 2022). Similar changes in cell wall composition have been observed in independently evolved succulents and are considered one of the features of the succulent syndrome (Fradera-Soler et al. 2022, 2023). Therefore, these genera are also well suited for comparative analyses exploring the relationship between cell wall characteristics and succulence. In future studies, systematic characterization of traits like leaf cell size and cell wall composition, including comparisons with closely related non-succulents, may be key to understanding complex succulent evolution. Several species within Asteraceae have been sequenced, and transformation techniques have already been established (Elomaa et al. 1998; Kishi-Kaboshi et al. 2017; Nakano et al. 2021). Thus, our findings highlight Asteraceae—particularly Crassothonna and related succulent taxa—as a promising model for investigating the mechanisms and evolutionary history of leaf succulence.

## Supplementary Information

Below is the link to the electronic supplementary material.

Supplementary Material 1

Supplementary Material 2

## Data Availability

The data that support the findings of this study are openly available in Dryad at https://datadryad.org/stash/share/ErhmVtdInCEK-F27AfZeSCPoAod4xVqJm02CBW9UQ4E.
