# Supplementary material for: Comparative analysis to investigate a possible mechanism for cell enlargement in succulent leaves of Crassothonna capensis (Asteraceae)
Source: J Plant Res. 2025 Nov 11;139(1):151–9. doi: 10.1007/s10265-025-01674-0 (PMC12868066; doi:10.1007/s10265-025-01674-0)
Supplement: Supplementary file 2 — Supplementary Material 2 [file 10265_2025_1674_MOESM2_ESM.pdf]

Supplementary Information

Title:
Comparative analysis to investigate a possible mechanism for cell enlargement in succulent leaves of *Crassothonna capensis* (Asteraceae)

Journal name:
Journal of Plant Research

Authors:
Hokuto Nakayama*, Kento Sawazaki, Yuki Doll, Hiroyuki Koga, Huibo Yu, Yasutake Moriyama, Mikita Tamura, and Hirokazu Tsukaya

Department of Biological Sciences, The University of Tokyo, Science Build. #2, 7-3-1 Hongo Bunkyo-ku Tokyo, 113-0033, Japan

*Correspondence:
Email: hokuto@bs.s.u-tokyo.ac.jp

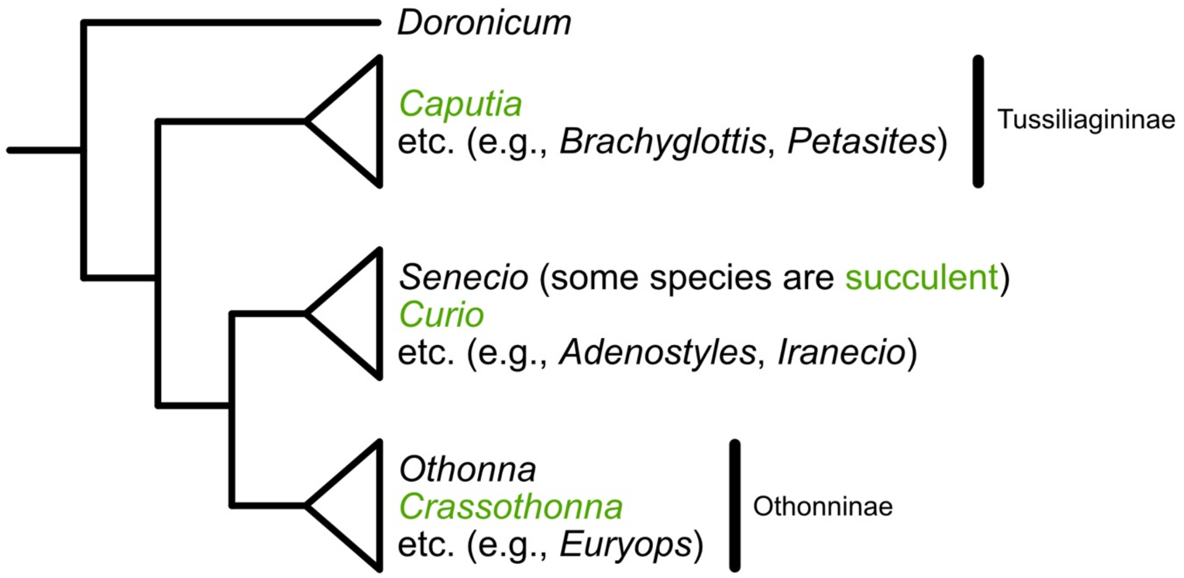

Fig. S1. Simplified phylogeny of plants used in this study.

A simplified tree shows the relationship between *Caputia, Crassothonna, Curio, Othonna,* and *Senecio* in Asteraceae. The tree was drawn based on multiple studies on phylogenies in Asteraceae (Cicuzza et al. 2018; Kandziora et al. 2016; Mandel et al. 2017; Pelser et al. 2010). Note that this is a rough phylogenetic tree. Because even though Othonninae seems to be rather well-resolved, and *Curio* seems to be a monophyletic, the genus *Senecio* is still rather unresolved.

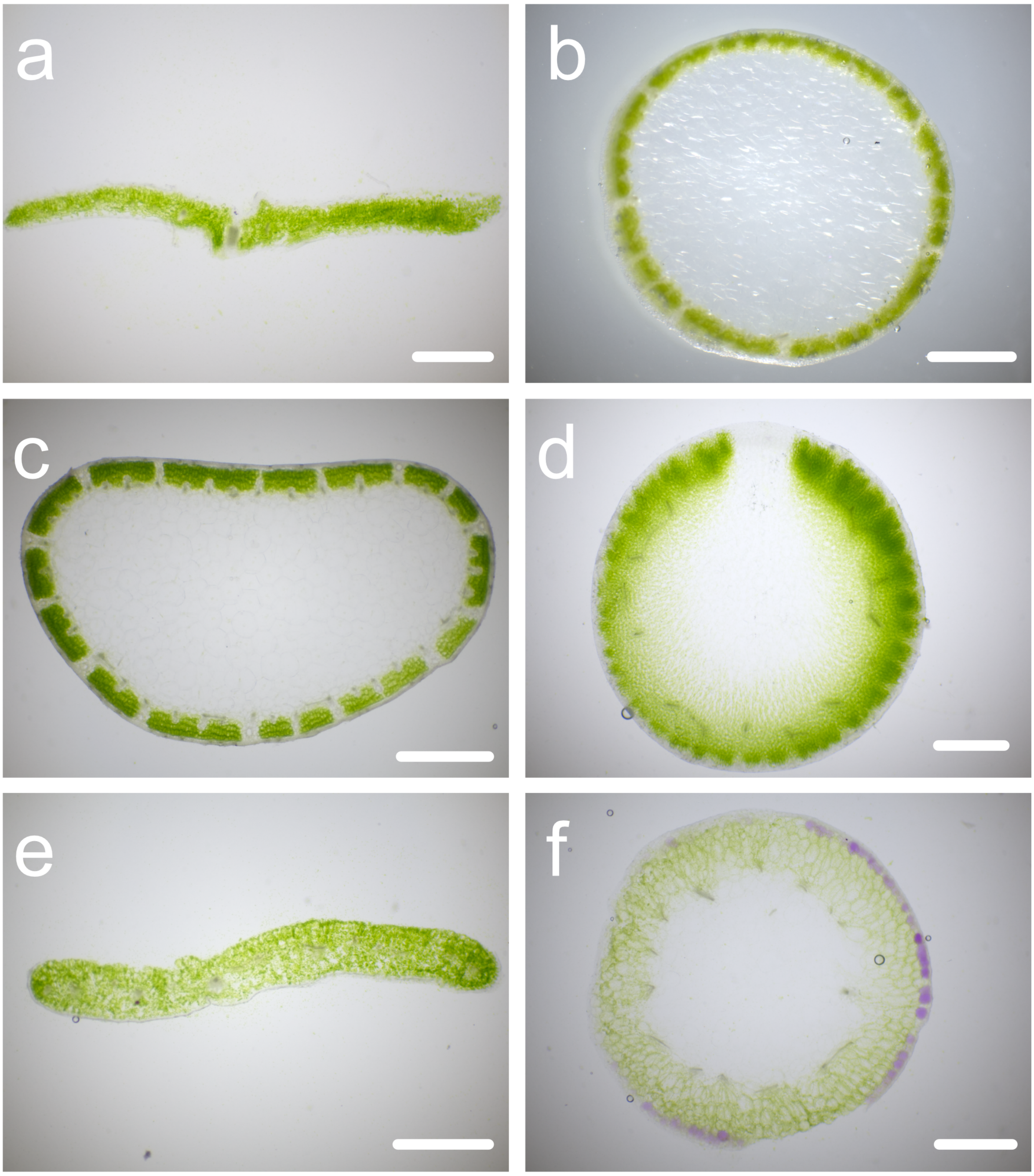

Fig. S2. Leaf cross sections of plants used in this study.

(**a**) *Senecio vulgaris*. (**b**) *Senecio antandroi.* (**c**) *Curio repens*. (**d**) *Curio rowleyanus*. (**e**) *Othonna euphorbioides*. (**f**) *Crassothonna capensis*. Bars = 1 mm.

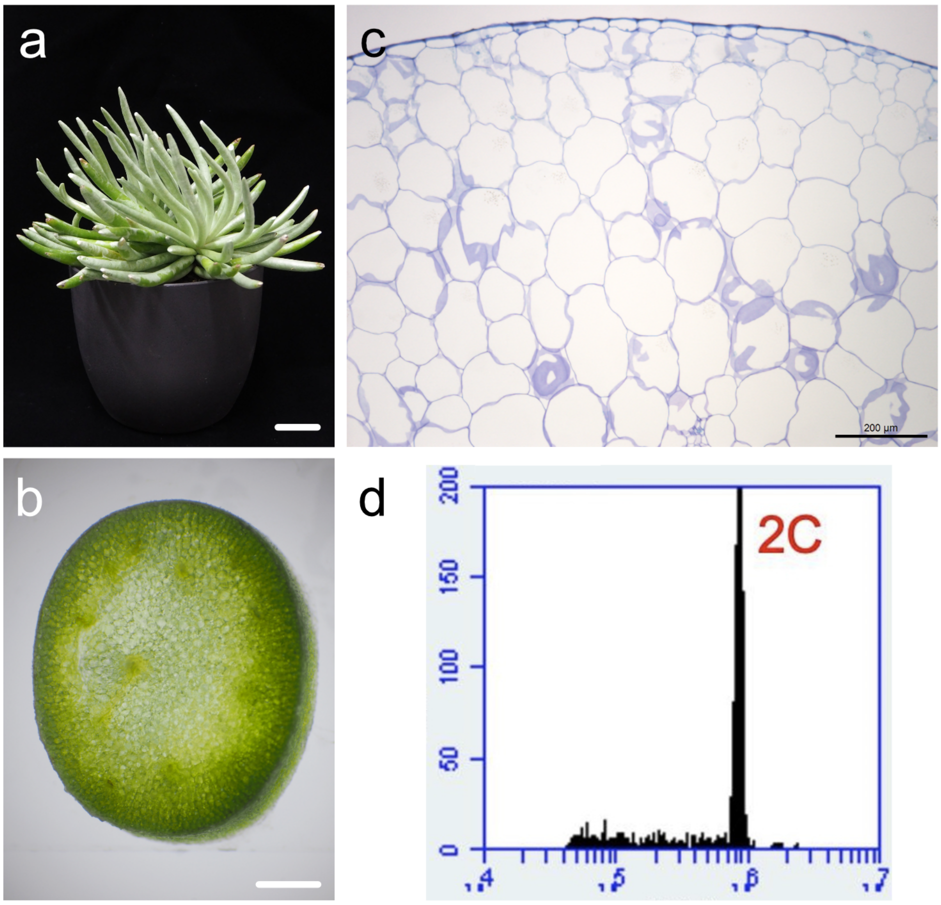

Fig. S3. Gross morphology, inner tissue anatomy, and nuclear ploidy distribution in leaves in *Caputia scaposa*.

(**a**) Side view of the shoot. (**b**) A leaf cross-section. (**c**) The inner morphology of a leaf. (**d**) Nuclear ploidy distribution in leaves. The x-axis indicates the signal intensity of propidium iodide, which reflects the nuclear DNA content, and the y-axis indicates the cell counts. Bars = 2 cm in (**a**), 1 mm in (**b**), and 200 µm in (**c**).
